# Real-Time Coupled Cluster Theory with Approximate Triples

**DOI:** 10.1021/acs.jpca.4c08499

**Published:** 2025-02-08

**Authors:** Zhe Wang, Håkon Emil Kristiansen, Thomas Bondo Pedersen, T. Daniel Crawford

**Affiliations:** † Department of Chemistry, Virginia Tech, Blacksburg, Virginia 24061, United States; ‡ Hylleraas Centre for Quantum Molecular Sciences, Department of Chemistry, University of Oslo, P.O. Box 1033 Blindern, N-0315 Oslo, Norway

## Abstract

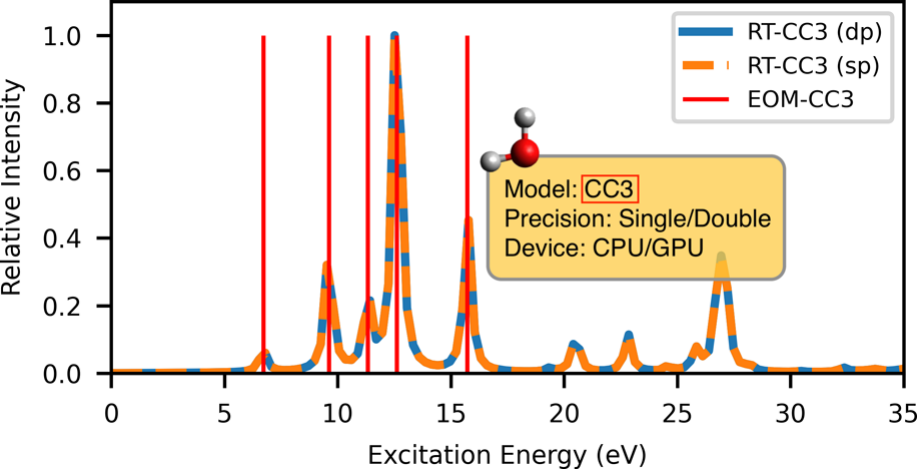

In order to explore the effects of high levels of electron correlation on the real-time coupled cluster formalism and algorithmic behavior, we introduce a time-dependent implementation of the CC3 singles, doubles, and approximate triples method. We demonstrate the validity of our derivation and implementation using specific applications of frequency-dependent properties. Terms with triples are calculated and added to the existing CCSD equations, giving the method a nominal (*N*^7^) scaling. We also use a graphics processing unit accelerated implementation to reduce the computational cost, which we find can speed up the calculation by up to a factor of 13 for test cases of water clusters. In addition, we compare the impact of using single-precision arithmetic compared to conventional double-precision arithmetic. We find no significant difference in polarizabilities and optical-rotation tensor results but a somewhat larger error for first hyperpolarizabilities. Compared to linear response CC3 results, the percentage errors of RT-CC3 polarizabilities and RT-CC3 first hyperpolarizabilities are under 0.1 and 1%, respectively, for a water-molecule test case in a double-ζ basis set. Furthermore, we compare the dynamic polarizabilities obtained using RT-CC3, RT-CCSD, and time-dependent nonorthogonal orbital-optimized coupled cluster doubles (TDNOCCDs) in order to examine the performance of RT-CC3 and the orbital-optimization effect using a set of ten-electron systems.

## 1 Introduction

Coupled cluster (CC) theory^1−5^ has been proven to be one of the most accurate and robust methods for treating electron correlation effects for a wide range of molecular properties, from ground-state energies and molecular structure to thermodynamic properties, electronic and vibrational spectra, response properties, and more. While CC methods are exact in the untruncated (Born-Openheimer, nonrelativistic) limit, restriction of the wave function to modest excitation levels is ultimately necessary for practical applications.^6^ While the inclusion of single and double excitations (CCSD) has been widely used and affirmed to be effective and efficient (with (*N*^6^) scaling, where *N* is related to the size of the system), higher levels are often required to achieve the accuracy needed for quantitative comparison to experiment. However, extension of the wave function even to include just full triples (CCSDT) is impractical for most applications due to the (*N*^8^) scaling of the method.^7,8^

Over the last several decades, researchers have explored a range of approximations to the full CCSDT model,^9−17^ the most successful of which is the CCSD(T) approach.^12^ In this approximation, the converged CCSD singles and doubles amplitudes are used to estimate the triples in a noniterative manner by adding dominant terms in the many-body perturbation theory (MBPT) expansion of the correlation energy. In particular, for Hartree–Fock reference determinants, the (T) correction includes triples contributions to the energy involving the doubles at fourth order and the singles at fifth order (which becomes fourth order for non-Hartree-Fock references). This leads to a noniterative (*N*^7^) method which is commonly referred to as the “gold standard” of quantum chemistry.

For response properties, such as dynamic polarizabilities, which require a time-dependent formulation, the CCSD(T) model suffers from the lack of coupling between the triples and the lower-excitation amplitudes. Thus, the triples do not respond directly to an external field, for example, yielding the same pole structure of the CCSD response functions. This was the motivation for the development of the CC3 approach by Koch, Christiansen, Jørgensen, and co-workers.^18,19^ CC3 is an iterative model that treats singles uniquely as zeroth-order parameters to approximate orbital relaxation effects, with triples being correct to the second order of MBPT. In each iteration, the approximate triples are calculated and used to determine their contributions to the singles and doubles, in order to correct the energy to fifth order. In this manner, contractions with a scaling of (*N*^7^) occur involving the triples amplitudes, but the complete triples tensor need not to be stored at any time during the calculation for a time-independent formulation.

CC3 can provide comparable results compared to other iterative and noniterative approximate triples models for time-independent properties.^19^ More crucially, CC3-level time-/frequency-dependent properties, including response functions, can be derived.^18−21^ If considered only in comparison with other iterative models, counting singles as zero-order parameters underscores the greater importance of singles for properties related to the perturbation of an external electromagnetic field and excited states. This makes it an exceptional candidate to be combined with, for example, linear- and high-order response theory,^22−25^ and real-time (RT) methods,^26−37^ in order to include higher excitations beyond CCSD. Implementations of CC3 response functions have been reported in comparison to other iterative triples models.^18^ For example, excitation energies of small molecules are significantly improved by CC3 relative to CCSD, and were found to be comparable to full CCSDT at greatly reduced cost.^38^ Second-order properties, such as polarizabilities, were also obtained from CC3 linear response functions and yielded good agreement with experimental data for the systems considered.^39^

Here, we report the first implementation of the RT-CC3 method, which is built upon our existing RT-CC framework.^36,37^ A similar RT-CC method with explicitly time-dependent orbitals and perturbation-based treatment of triple excitation amplitudes was presented recently by Pathak et al.^40,41^ We have computed RT-CC3 absorption spectra for several small molecular test cases for comparison to excitation energies and dipole strengths obtained from conventional time-independent response theory to validate the RT-CC3 implementation. For higher-order properties, instead of using the derivatives of the time-averaged quasienergy, i.e., a response formulation, we have used time-dependent finite-difference methods^42^ to obtain polarizabilities, the *G′* tensor related to optical rotation, first hyperpolarizabilities, and the quadratic response function ⟨⟨***m̂***;**μ̂**, **μ̂**⟩⟩_ω,ω′_. This approach was first proposed by Ding et al. in an application of real-time time-dependent density functional theory (RT-TDDFT), allowing response properties to be obtained from a cohort of propagations using different (weak) field strengths.^43^ We find that only relatively short propagations are required to obtain such properties and that properties related to different orders of response to the same field can be calculated using the same group of propagations. We provide details regarding our implementation of the RT-CC3 method, the accuracy and stability of the corresponding simulations, as well as discussion of the finite-difference methods and comparison with RT simulations at other levels of theory in subsequent sections.

## 2 Theory

### 2.1 Implementation of the RT-CC3 Method

The Hamiltonian perturbed by an external field can be defined as
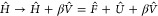
1where  is the Fock operator,  is the fluctuation potential, β is the field strength, and  is the one-electron perturbation operator, such as an electromagnetic field, with strength, β. In RT-CC methods, the differential equations of the time-dependent  and  amplitudes can be derived from the time-dependent Schrödinger equation by explicitly differentiating the amplitudes with respect to time, viz.,
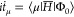
2and
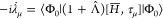
3where  is the similarity-transformed Hamiltonian,, and τ_μ_ is the second-quantized operator that produces the excited determinant |μ⟩ from the reference |Φ_0_⟩. It is important to note that the right-hand sides of the two equations are equivalent to the amplitude residuals in the ground state amplitude equations. Consequently, the computational cost of evaluating the right-hand side once is equivalent to the cost of calculating the corresponding amplitude residual during a single iteration of the ground-state calculation. The amplitudes are inherently complex-valued functions of time.

The complete derivation and the spin-adapted expression of the RT-CC3 equations are provided in the Appendix, while key details of the implementation are highlighted here. For RT-CC3 calculations, amplitude residuals of singles and doubles can be separated into the CCSD component and the contribution from the triples. In each time step of the real-time propagation, the CCSD amplitude residuals are calculated first, followed by the calculation of the contribution from triples to singles and doubles. Since the *T̂*_3_ amplitude is a six-index quantity, storing the entire tensor with a size of  is neither preferable nor feasible due to limited memory, especially when dealing with large molecular systems and/or large basis sets. The treatment of triples differs depending on wether the external perturbation is present or absent during the calculation.

Given the general form of CC3  and  equations
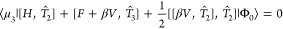
4and
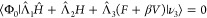
5respectively, the terms can be rearranged as
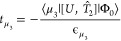
6and
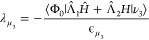
7when the perturbation is absent (β = 0). *H*, *F*, *U*, *V* are the *T*_1_-transformed Hamiltonian, Fock operator, fluctuation operator, and perturbation operator, respectively, with a *T*_1_-transformed operator defined as
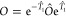
8Any subset of triples can be calculated explicitly with singles and doubles. During each time step, only a specific subset of triples is calculated on-the-fly when it is needed in a contraction. For example, in the spin-adapted  equation, the contribution of triples can be calculated as
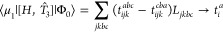
9The subset of triples corresponding to a certain set of occupied orbitals *i*, *j*, *k* is calculated and contracted with the subset of integrals corresponding to the same orbitals *j* and *k* to calculate its contribution to the  amplitudes with the occupied orbital *i*. It is important to note that the subset of triples to be calculated can be a tensor with fixed occupied orbitals *i*, *j*, *k*, or fixed unoccupied orbitals *a*, *b*, *c*. The former approach requires performing the triples calculations and subsequent contractions  times, while the latter requires these calculations  times. As *N*_*V*_ is typically much larger than *N*_*O*_, it is typically more efficient to compute  blocks of triples for a given *i*, *j*, *k* combination than to compute  blocks for a given *a*, *b*, *c* combination.

When the perturbation is present, the terms ⟨μ_3_|[β*V*, ]|Φ_0_⟩ and  in eq 4, and ⟨Φ_0_| (β*V*)|ν_3_⟩ in eq 5 must be included. In such cases, the complete set of triples must be calculated for the terms involving *T̂*_3_ and Λ̂_3_. For the efficency of the calculation, the triples are calculated before their first usage in an evaluation of the amplitude residual and are kept in memory or on disk until the end of the evaluation. Thus, the calculation requires larger storage space, which could be a critical limitation for large molecules and/or basis sets. The calculation of triples and the *V*-dependent terms will also lead to an increased running time. Similarly, when calculating the one electron density matrix, the triples are computed once and shared for different blocks of the matrix. For the real-time propagation, the *V*-dependent terms do not always need to be calculated throughout the whole propagation. If the external field is switched off at a certain time step, the full set of triples and the additional terms in the triples equations are no longer needed for the rest of the propagation.

### 2.2 Frequency-Dependent Properties from RT Simulations

#### 2.2.1 Absorption Spectrum

For electromagnetic fields in the dipole approximation, the perturbation operator can be specifically written as
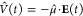
10with the system interacting with an external electric field **E**(*t*). Linear absorption spectra can be calculated using the frequency-dependent counterparts of the time-dependent dipole and electric field, obtained via the Fourier transform:
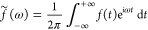
11The dipole strength function used here to quantify the probability of the absorption process is proportional to the imaginary part of the trace of the dipole polarizability tensor **α**(ω),
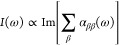
12where the subscript β is the Cartesian axis *x*, *y*, *z*. The dipole polarizability α_ββ_ can be calculated as
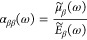
13

#### 2.2.2 Dynamic Polarizabilities and Hyperpolarizabilities

Consider a molecule exposed to a field with the form of
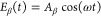
14where *A*_β_ and ω are the maximum amplitude and the frequency of the field, respectively, with the subscript β being the Cartesian axis that indicates the direction of the field. Under this electric field, the time-dependent electric dipole moment can be expanded as (see, e.g., refs (43) and (44) for details)
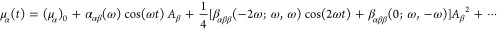
15where α(ω) is the polarizability and β(−2ω; ω, ω) and β(0; ω, –ω) are the first-hyperpolarizabilities corresponding to the second-harmonic generation (SHG) and optical rectification (OR), respectively. Alternatively, if we write the series expansion of the electric dipole moment as
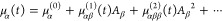
16and then equate eqs 15 and 16, we obtain
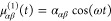
17and
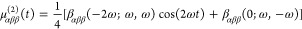
18One way to calculate the first- and second-order dipole moments is using the (central) finite-difference method, which is commonly employed for numerical differentiation. To apply it to real-time methods, induced dipole moments from simulations with different field strengths are required. For instance, conducting four separate simulations with field strengths of *A*, −*A*, 2*A*, and −2*A* as the only varying parameter, allows us to express  and  as
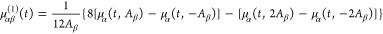
19and
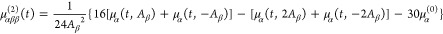
20With the value of  at each time step, we can fit the trajectory to a cosine curve, as shown in eq 17. The polarizability α(ω) will be the amplitude of the fitted curve. Similarly, the trajectory of  can also be fitted to a curve with the form 1/4[*A* cos(ω*t*) + *B*], where β(−2ω; ω, ω) and β(0; ω, – ω) are the values of *A* and *B* respectively. Additional details about the finite difference method and its application in the real-time framework can be found in refs (43) and (44). It is worth noting that although calculating (hyper-)polarizabilities at each frequency requires four real-time simulations, each simulation does not need to be as long as the ones used for calculating the absorption spectrum, where the spectral resolution inherently depends on the propagation length. Moreover, both polarizabilities and hyperpolarizabilities at the same frequency can be obtained from the same set of simulations. The difference lies only in the postprocessing steps.

#### 2.2.3 *G′* Tensor and Magnetic-/Electric-Dipole Quadratic Response Function

In addition to the properties associated with the induced electric dipole moments, the *G′* tensor that is related to linear chiroptical properties (optical rotation, electronic circular dichroism, etc.) and the response function ⟨⟨*m̂*_α_; μ̂_β_, μ̂_β_⟩⟩ are also accessible under this formalism, in that they are connected to the magnetic dipole moments induced by external electric fields. Following the same steps as above, we first write the time series expansion of the magnetic dipole moment as
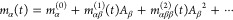
21For the *G′* tensor corresponding to the first-order induced magnetic dipole moments, we expand *m*_α_ as
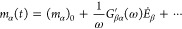
22with the time derivative of the field being *Ė*_β_ = −*A*ω sin(ω*t*), and then equate it with eq 21.  can thus be written as
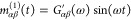
23and calculated as
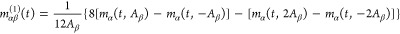
24To obtain ⟨⟨; , ⟩⟩ from the second-order induced magnetic dipole moments, we expand *m*_α_ in the frequency domain alternatively as
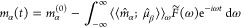
25
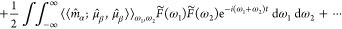
26By equating eq 26 and eq 21,  can then be written as
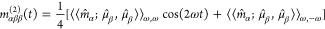
27and calculated as
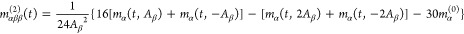
28

For the magnetic dipole moments, no additional modifications to the real-time framework are needed. Once we calculate the one-electron density, the electric dipole moment can be obtained by contracting the density with the electric dipole operator, and the magnetic dipole moment can be obtained by contracting the density with the magnetic dipole operator. It is worth mentioning that the simple contraction of the density with the magnetic dipole matrix elements is not gauge-invariant since the orbitals are fixed in our implementation.

#### 2.2.4 Ramped Continuous Wave

As shown in eq 14, a cosine wave with a frequency of ω is used to calculate the optical properties. In practice, instead of having the same field from the beginning to the end, a ramped wave is applied, gradually switching on the field. There are two types of ramped waves that are typically used, a linear ramped continuous wave (LRCW)^43^
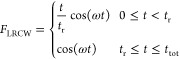
29and a quadratic ramped continuous wave (QRCW)^44^
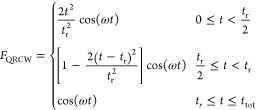
30where *t*_r_ is the duration of the ramped field and *t*_tot_ is the total length of the simulation. For a field with a frequency ω, an optical cycle given by
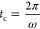
31With *n*_r_ and *n*_p_ the number of optical cycles used for ramping and for subsequent propagation at full field strength, the total propagation time becomes
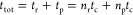
32Here, *t*_p_ denotes the portion of the propagation utilized for property fitting. Ofstad et al. demonstrated in ref (44) that the QRCW can reduce the number of optical cycles required for both the ramped and subsequent cycles. This reduction is attributed to the QRCW’s more gentle amplification over time in comparison to the LRCW, resembling an adiabatic switch-on of the field. Moreover, the QRCW is smooth whereas the LRCW has discontinuous first derivatives at the start and end points of the ramp. The QRCW thus allows the system to stabilize more rapidly, even in a shorter time, ensuring that the electrons do not experience an abrupt perturbation initially. Ofstad et al. concluded that for accurate fitting of polarizabilities and first hyperpolarizabilities, one ramped cycle and one subsequent cycle for curve fitting are sufficient, providing accurate results compared to linear response theory, which assumes a monochromatic pulse that is adiabatically switched on by definition. They also demonstrated through multiple test cases that the LRCW typically requires at least four subsequent cycles following a single ramped cycle to achieve convergence and accuracy. Thus, in the present work, we have carried out RT-CC3 calculations with *n*_r_ = *n*_p_ = 1 as the default values for the QRCW, and *n*_r_ = 1, *n*_p_ = 4 as the default values for the LRCW in comparison.

## 3 Computational Details

When calculating the absorption spectrum and comparing it with equation-of-motion CC (EOM-CC) results, an isotropic electric field shaped as a Gaussian function is applied to the system and shown as
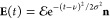
33where the vector **n** represents the direction of the field as
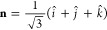
34The center ν and the width σ of the field are chosen to be 0.01 and 0.001 au, respectively, to mimic a delta pulse that is switched on at the beginning of the propagation. For the calculation of RT-CC3/cc-pVDZ absorption spectrum of H_2_O, the field strength , step size *h*, and propagation time *t*_f_ were chosen to be 0.01, 0.01, and 300 au, respectively. Padé approximants^45^ were used to improve the resolution of the RT-CC3 spectrum.

To further test the performance of our RT-CC3 implementation, CPU and GPU calculations were carried out using water monomer, dimer, and trimer systems in both single- and double-precision. Each CPU calculation was run on a single node with an AMD EPYC 7702 chip, and each GPU calculation was run on a single node with an Nvidia Tesla P100 GPU. Tensor manipulation was conducted using NumPy^46^ and PyTorch^47^ for the CPU and GPU calculations, respectively, with similar syntax. Tensor contraction was performed using opt_einsum,^48^ and a PyTorch backend was specifically employed for the GPU calculation. All calculations kept the 1*s* orbitals of the oxygen atoms frozen.

For calculating dynamic polarizabilities and first hyperpolarizabilities, a set of RT calculations for the water molecule with the cc-pVDZ basis set^49^ were executed using field strengths of 0.002, −0.002, 0.004, and −0.004 au at both the CC3 and CCSD levels. The step size was set to 0.01 au. The carrier frequency of the field was set to 0.078 au, which corresponds to a wavelength of 582 nm and is lower than the resonance at 0.247 au for the water molecule. The molecule was subjected to a field in the *x*, *y*, and *z* directions individually to obtain the corresponding elements of the polarizabilities and first hyperpolarizabilities tensors. For the *G′* tensors and the response function ⟨⟨; , ⟩⟩, the same electric field was applied to the H_2_ dimer with the cc-pVDZ basis set. The *G′* tensor elements and the response functions were calculated from the induced magnetic dipole moments. The frequency of 0.078 au is below the resonance at 0.367 au. Curve fitting was performed using scipy.optimize.curve_fit.^50^ All calculations were performed on a single Nvidia Tesla P100 GPU, and both single- and double-precision calculations were conducted and compared. The results from the RT simulations were also compared to reference values obtained from the Psi4^51^ and CFOUR^52^ packages.

RT-CC methods were also compared to the time-dependent nonorthogonal orbital-optimized coupled cluster doubles (TDNOCCD) method^53^ for calculating polarizabilities with ten-electron systems including Ne, HF, H_2_O, NH_3_, and CH_4_. The field strengths of the propagations were chosen to be 0.001, −0.001, 0.002, and −0.002 au. Various frequencies below the resonance of the corresponding molecule were tested. The basis set was chosen to be aug-cc-pVDZ^54^ for HF, H_2_O, NH_3_, and CH_4_, and d-aug-cc-pVDZ^55^ for Ne. The QRCW method was utilized for accuracy and efficiency. The length of each propagation is two optical cycles, depending on the frequency of the field. The time step of the propagations was 0.01 au. All calculations were performed in double-precision.

All calculations were run in PyCC^56^ with the stationary electric and magnetic dipole operators extracted from Psi4. The Runge–Kutta fourth-order integrator^57^ was used for the RT propagations. For the series of water clusters, (H_2_O)_*n*_ up to *n* = 4, used in Section 4.1, as well as for H_2_O in Section 4.2.2, the geometries were provided by Pokhilko et al.^58^ Five ten-electron systems, Ne, HF, H_2_O, NH_3_, and CH_4_, were taken as test cases in Section 4.2.2, using geometries provided by Kristiansen et al.^59^ The geometries of the H_2_ dimer for the *G′* tensor calculation in Section 4.2.3 can be found in the dictionary of molecular structures of PyCC. All geometries are also available in the Supporting Information (SI).

## 4 Results and Discussion

### 4.1 Computational Cost of the RT-CC3 Method

The CC3 method scales as (*N*^7^), making it significantly more expensive than the CCSD method. In the implementation, techniques including factorization, reordering, and memory management need to be considered to improve efficiency, while the scaling remains unchanged. This is standard practice in quantum chemistry and is expected when dealing with such steeply scaling methods, and has been extensively discussed with respect to the CC3 method, in particular, in the literature.^18,19,60^ Taking the contribution from triples to the  equation shown in eq A23 as an example, several adjustments can be made to accelerate the calculation. For contractions involving three tensors, an intermediate consisting of two tensors is calculated first to avoid a  contraction. The selection of the two tensors in the initial step may also affect efficiency. For instance, we can rewrite the third term in eq A23 in two alternatives:
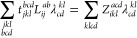
35where
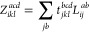
36or
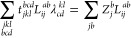
37where
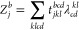
38The former approach results in a scaling of (*N*^6^), whereas the intermediate approach requires a contraction that scales as (*N*^8^). The latter approach results in a scaling of *N*^4^ with an intermediate contraction of (*N*^6^), making it the favorable way to calculate this specific term. The second term in eq A23 can be rewritten as
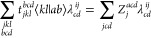
39where
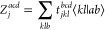
40or
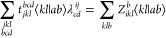
41where
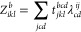
42In this case, the two alternatives share the same scaling of (*N*^7^) for the contraction and (*N*^5^) for the calculation of the intermediates. The former factorization results in a scaling of , while the latter one results in a scaling of . Following the same approach as was done for the third term in the equation, the latter method should be preferable since *N*_*V*_ is usually larger than *N*_*O*_ and grows faster when a larger basis set is used. However, it is important to note that the intermediate  in eqs 39 and 40 does not contain any Λ̂ amplitudes, and thus it can be calculated before the iterations and only needs to be computed once during the ground state calculation. Similar considerations are taken into account for the other terms in the CC3 equations as well.

Another computationally expensive step in the RT-CC3 method is the calculation of the occupied-occupied block of the one-electron density, as shown in eq A31. This calculation involves the contraction of the  and  amplitudes, which only differ in one index corresponding to the occupied orbital. Nested loops over virtual orbitals are required for this calculation. For the density matrix elements, we choose to implement a two-layer nested loop over virtual orbitals, considering it as a four-index quantity for the triples with two fixed virtual orbitals. This is preferred over a three-index quantity approach with three fixed virtual orbitals. The contraction can be written as
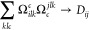
43for a certain pair of virtual orbitals *a*, *b*. Reducing the number of loops over virtual orbitals accelerates the calculation of the one-electron density substantially. For the ground state calculation, the density needs to be calculated only once after the amplitudes converge from the iterations. However, for RT simulations the acceleration of the density calculation is particularly important because it is carried out in every time step to obtain the corresponding time-dependent properties.

In addition to the above, the permutational symmetry of the amplitudes shown in eqs A29 and A30, as well as the permutational symmetry of the integrals, are facilitated in both derivation and implementation. Identical terms that only differ in ordering need to be identified to avoid repeated calculation with a polynomial scaling. Regarding the triples, the amplitudes contracted with the same integral or other amplitudes should be reordered first. For instance, in eq A21,  amplitudes contribute to  amplitudes by contracting with two-electron integrals. Two distinct triples are required in the contraction. Instead of calculating two  amplitudes individually, the amplitudes are reordered so that they share the same set of occupied orbitals. Given the known  with a fixed set of *i*, *j*, *k*,  can be obtained simply by swapping the first and third axis of the 3-index quantity. As noted by Paul et al.,^60^ the computational time for reordering can be significant depending on the system size and the hardware used for the calculation. Nevertheless, the calculation of an additional set of triple amplitudes is still much more expensive and thereby dominant in the computational cost in our implementation. Additionally, it is worth noting that the permutational symmetry of the -transformed integrals is reduced relative to the untransformed integrals: swapping both pairs of indices in the bra and ket, viz.,
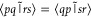
44

To assess the performance of our RT-CC3 implementation, we determined the computational time for each time step, as shown in Table 1 for the water monomer, dimer, trimer, and tetramer. Using the cc-pVDZ basis set, a single water molecule has 5 occupied orbitals (*N*_*O*_) and 19 virtual orbitals (*N*_*V*_). Each calculation was conducted exclusively on a single node to ensure consistency in computational resources. All contractions were done on either a CPU or a GPU.

**Table 1:** Performance Comparison of RT-CC3/cc-pVDZ Calculations for Water Clusters Using Different Hardwares and Precisions: Double-Precision on the CPU (CPU-dp), Single-Precision on the CPU (CPU-sp), Double-Precision on the GPU (GPU-dp), and Single-Precision on the GPU (GPU-sp)^a^

water cluster	*t*_CPU-dp_	*t*_CPU-sp_	*t*_GPU-dp_	*t*_GPU-sp_	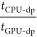	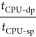	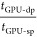
monomer	16.105	11.192	18.511	18.661	0.86980	1.4390	0.99196
dimer	814.94	410.92	256.95	259.31	3.1716	1.9832	0.99090
trimer	10743	5364.1	806.52	768.49	13.320	2.0028	1.0495
tetramer			2455.7	1981.8			1.2391

a Timings (first four columns) are reported in seconds as per-step averages over five time steps. The final three columns indicate speed-ups, calculated as ratios of timings for each case.

When transitioning from the monomer to the trimer, the system size increases by a factor of 3, theoretically causing the computational time to rise by a factor of 3^7^ ≈ 2200. As shown in the table, the CPU-dp calculation for the water trimer takes approximately 3^5.92^ times longer than the monomer, while the running time of the CPU-sp calculation increases by around 3^5.62^. For the GPU calculations, the increase from the monomer to the trimer is approximately 3^3.44^ for the double-precision calculation and 3^3.38^ for the single-precision case. Furthermore, the GPU-dp calculation for the tetramer takes about 4^3.53^ times longer than the monomer, while the single-precision calculation takes approximately 4^3.36^ times longer. As the system size continues to grow, the scaling will eventually reach (*N*^7^) as defined.

It is evident that the application of single-precision does not achieve the ideal doubling of the calculation speed, especially for GPU implementations. Nevertheless, the speedup from CPU-sp becomes more noticeable as the system size increases, and a discernible speedup emerges for GPU-sp calculations when the system size reaches 96 molecular orbitals. Additionally, a considerable speedup was attained from the GPU implementation overall. For the water trimer, the GPU-dp calculation is 13 times faster than the CPU-dp calculation. We anticipate further speedups from GPUs for even larger systems until a memory limitation is encountered. It is also worth mentioning that our GPU implementation utilizes the speedup of tensor contractions on GPUs, however, it is not fully optimized in terms of memory allocation, parallelization, and other factors.

### 4.2 Optical Properties

#### 4.2.1 Absorption Spectrum

**Figure 1:**
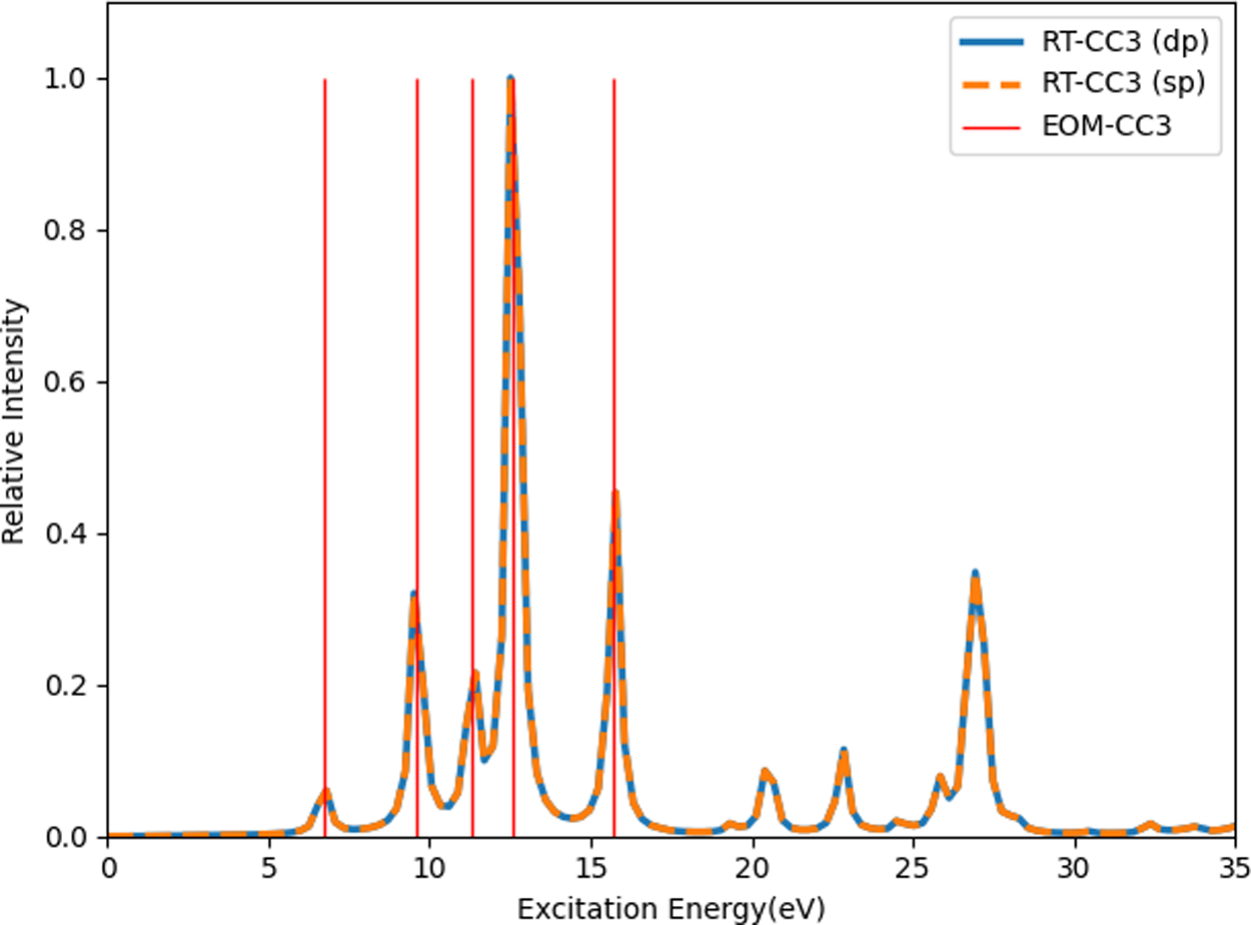
RT-CC3/cc-pVDZ linear absorption spectrum of H_2_O with vertical lines indicating the corresponding EOM-CC3/cc-pVDZ excitation energies.

To assess the stability and accuracy of the RT-CC3 implementation, we initially calculated the linear absorption spectrum using the procedure outlined in Section 2.2.1. To generate a broadband spectrum, a thin Gaussian pulse was applied. The absorption spectrum was computed for both single- and double-precision arithmetics, as depicted in Figure 1. It has been demonstrated that single-precision is sufficient for calculating the absorption spectrum using RT-CCSD in our previous work.^36^ Similarly, in this specific test case, no significant distinction between single- and double-precision results is discernible in the RT-CC3 outcomes. For the EOM-CC3/cc-pVDZ calculation, only excitation energies are attainable from Psi4, while the corresponding oscillator strengths remain unavailable. For illustrative purposes, the “height” of the stick spectra is chosen to be 1 to enable convenient visualization of the position of each state. However, this choice does not convey any information about the probability of the corresponding transition. Through this comparison, we can ascertain that the RT-CC3 method aligns well with the EOM-CC3 method.

#### 4.2.2 Dynamic Polarizabilities and First Hyperpolarizabilities

**Figure 2:**
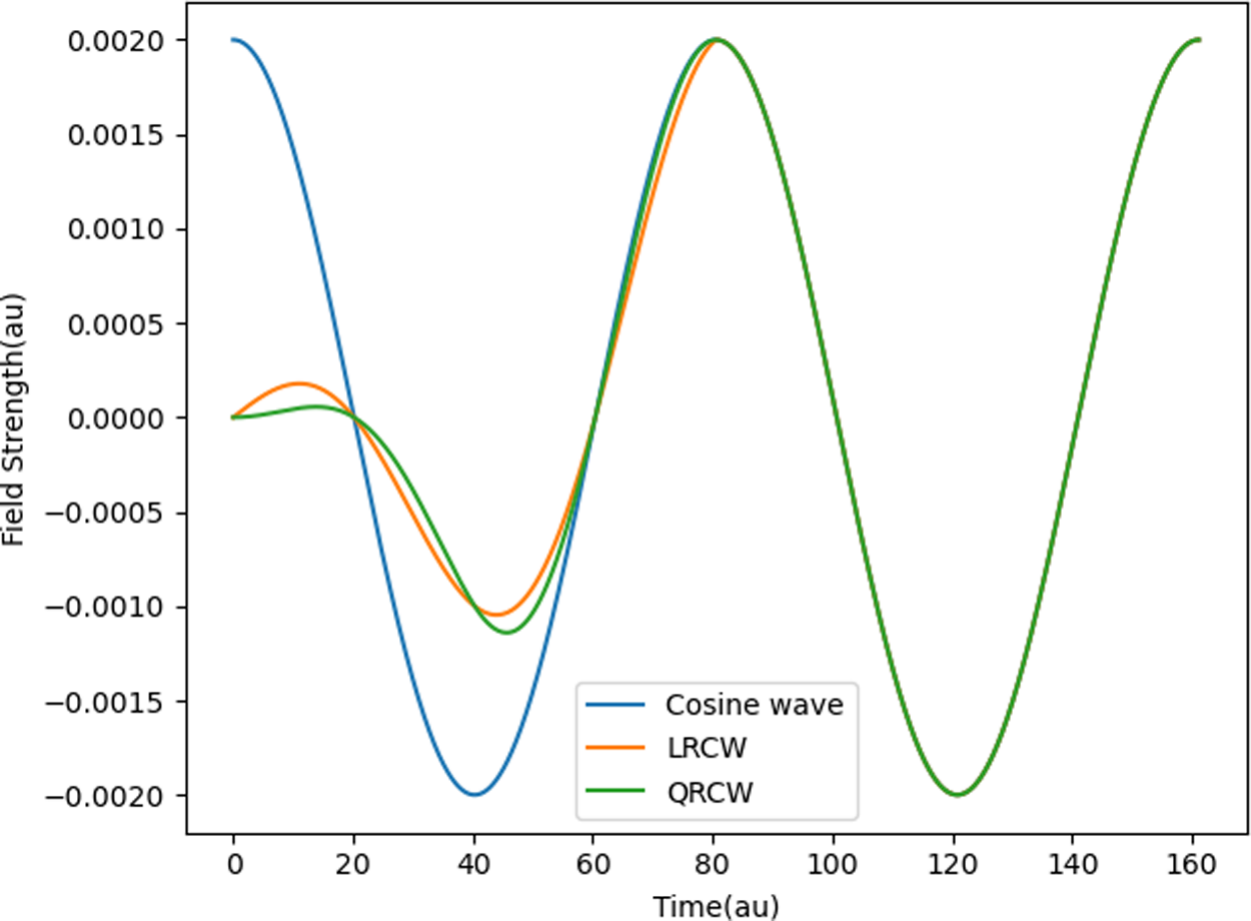
LRCW and QRCW of two optical cycles. Both of the RCWs have one ramped cycle following a cycle with a regular cosine wave. The frequency and the field strength are 0.078 and 0.002 au, respectively.

As demonstrated in ref (44), the QRCW is favorable for extracting optical properties as it has a smoother switch-on compared to the LRCW or a simple oscillatory field without ramping. Figure 2 illustrates that both LRCW and QRCW have significantly smaller amplitudes at the initial stages of the simulation compared to the regular cosine wave. Compared to LRCW, the QRCW curve exhibits a more gradual increase during the first 20 au and more closely follows the cosine curve during the final 20 au of the ramping stage. We apply both the LRCW and the QRCW to showcase the effect of ramping.

**Figure 3:**
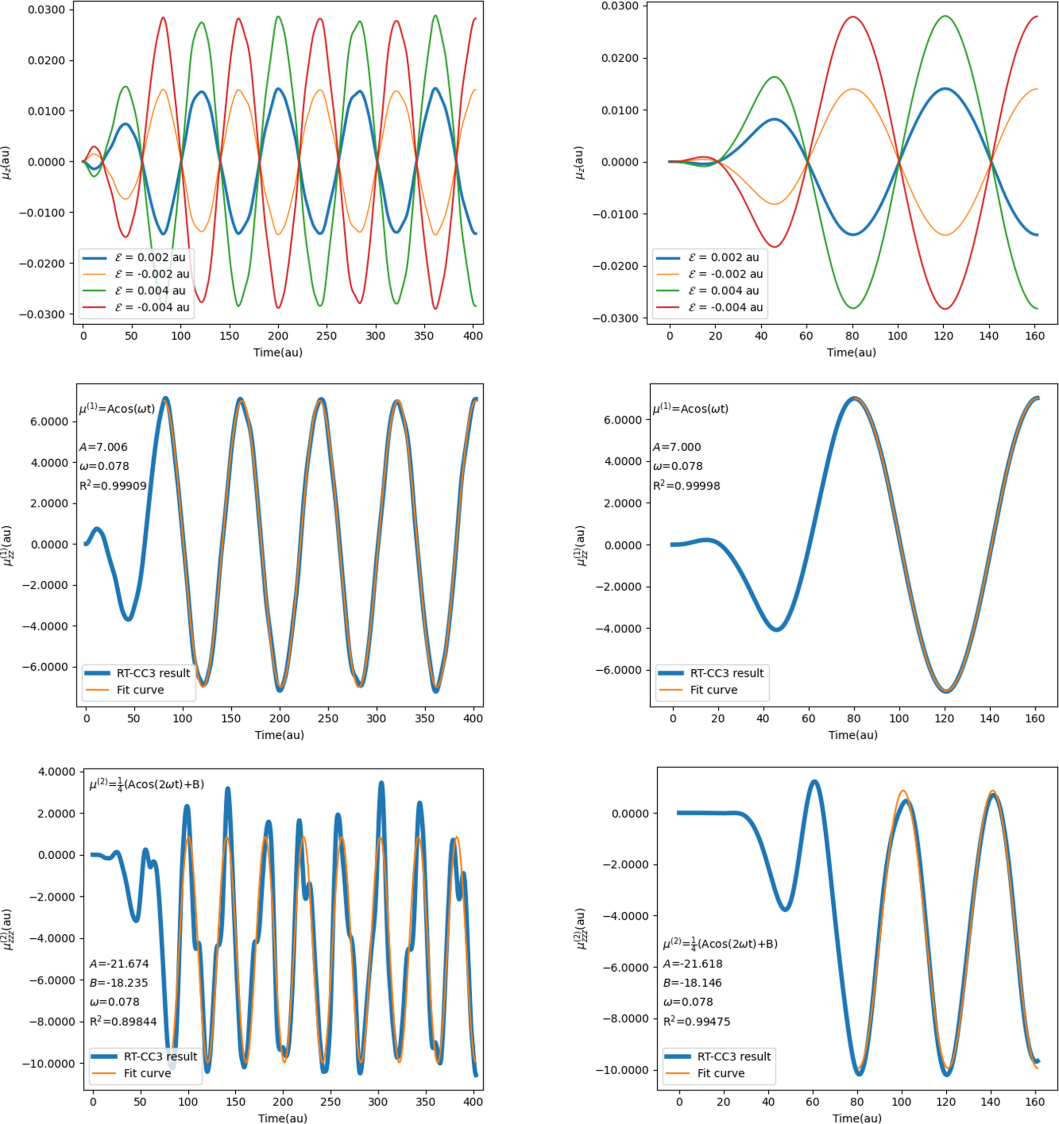
RT-CC3/cc-pVDZ (dp) results for the *z*-component of the induced dipole moment of H_2_O from simulations with field strengths ±0.002 au and ±0.004 au. The left column displays the LRCW results with *n*_r_ = 1 and *n*_p_ = 4 for the total dipole moment (top), the first- (middle) and second-order (bottom) dipole moments, including the curves obtained by fitting. The right column showcases the QRCW results with *n*_r_ = *n*_p_ = 1.

Dynamic polarizabilities and first hyperpolarizabilities of H_2_O at the level of CCSD and CC3 are calculated using finite-difference methods. The LRCW simulation spans five optical cycles, with the first cycle reserved for linear ramping. In contrast, the QRCW simulation encompasses only two optical cycles, again with ramping applied in the first cycle. The calculations are conducted using both single-precision (sp) and double-precision (dp) arithmetic. A representative result of RT-CC3/cc-pVDZ (dp) for H_2_O is depicted in Figure 3 to elucidate the procedure for obtaining polarizabilities and first hyperpolarizabilities.

From the fitted curve of the time trajectory of the first-order dipole moment, the corresponding polarizability component can be calculated as the amplitude of the curve. As depicted in Figure 3, the values of α_*zz*_ at ω = 0.078 au are 7.006 and 7.000 au, respectively, when utilizing the LRCW and QRCW. Regarding the first hyperpolarizabilities, the time trajectory of the second-order dipole moment is fitted into a cosine curve, determining the amplitude *A* and the phase *B*, which represent the hyperpolarizabilities associated with SHG and OR, respectively. The quality of the curve fitting is assessed using the *R*^2^ value. As shown in Figure 3, a well-fitting curve is characterized by an *R*^2^ value close to one, whereas a relatively inadequate fitting due to an irregular-shaped second-order dipole trajectory is indicated by an *R*^2^ value as low as 0.89839. The summarized results are presented in Tables 2, 3, and 5.

**Table 2:** RT-CCSD/cc-pVDZ and RT-CC3/cc-pVDZ Polarizabilities (in Atomic Units) of H_2_O at 582 nm from Simulations with Linear Ramped Continuous Wave (LRCW) or Quadratic Ramped Continuous Wave (QRCW) Fields^a^

	method	α_*xx*_	α_*yy*_	α_*zz*_			
	LR-CCSD	3.182	10.549	7.017			
LRCW	RT-CCSD (dp)	3.183	10.549	7.019	0.99980	0.99994	0.99909
	RT-CCSD (sp)	3.183	10.549	7.019	0.99980	0.99994	0.99909
QRCW	RT-CCSD (dp)	3.182	10.549	7.014	0.99999	0.99994	0.99998
	RT-CCSD (sp)	3.182	10.549	7.014	0.99999	0.99994	0.99998
	LR-CC3	3.164	10.581	7.007			
LRCW	RT-CC3 (dp)	3.166	10.577	7.006	0.99981	0.99993	0.99909
	RT-CC3 (sp)	3.166	10.577	7.006	0.99981	0.99993	0.99909
QRCW	RT-CC3 (dp)	3.164	10.576	7.001	0.99999	0.99994	0.99998
	RT-CC3 (sp)	3.164	10.576	7.001	0.99999	0.99994	0.99998

a Reference values from LR-CCSD and LR-CC3 calculations using CFOUR are provided for comparison. The *R*^2^ values, indicating the quality of curve fitting, are presented in the last three columns.

**Table 3:** RT-CCSD/cc-pVDZ and RT-CC3/cc-pVDZ First Hyperpolarizabilities (in Atomic Units) Associated with Second Harmonic Generation (SHG) of H_2_O at 582 nm Obtained from Simulations with Linear Ramped Continuous Wave (LRCW) and Quadratic Ramped Continuous Wave (QRCW) Fields^a^

	method	β_*zxx*_	β_*zyy*_	β_*zzz*_			
	LR-CCSD	–4.091	–35.441	–22.423			
LRCW	RT-CCSD (dp)	–4.311	–35.694	–22.485	0.93021	0.54362	0.89911
	RT-CCSD (sp)	–4.298	–35.707	–22.482	0.92954	0.54195	0.89921
QRCW	RT-CCSD (dp)	–4.053	–35.469	–22.435	0.99892	0.97345	0.99488
	RT-CCSD (sp)	–3.987	–35.520	–22.447	0.99169	0.97266	0.99480
LRCW	RT-CC3 (dp)	–4.081	–35.675	–21.674	0.92891	0.59673	0.89844
	RT-CC3 (sp)	–4.095	–35.686	–21.667	0.92874	0.59542	0.89839
QRCW	RT-CC3 (dp)	–3.828	–35.414	–21.618	0.99887	0.97110	0.99475
	RT-CC3 (sp)	–3.820	–35.379	–21.611	0.99273	0.97154	0.99474

a Reference values from LR-CCSD calculations using CFOUR are provided. The *R*^2^ values, reflecting the quality of curve fitting, are displayed in the last three columns.

To assess the performance of different simulations, three criteria are evaluated: (1) accuracy compared to linear response (LR) CC, (2) *R*^2^ value, and (3) simulation length. A method capable of delivering accurate results from a relatively short simulation, along with a curve fitting that yields a high *R*^2^ value, is the preferable choice. We employ the percentage error to quantify accuracy, which is calculated using the following formula:
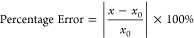
45where *x* is the measured value and *x*_0_ is the reference value.

For polarizabilities, the single- and double-precision calculations yield identical results up to three decimal places, with the same *R*^2^ values accurate for five decimal places. Minor discrepancy can be observed when comparing to the LRCW and the QRCW results. In RT-CCSD simulations, the LRCW results exhibit a 0.03% error in α_*xx*_ and a 0.03% error in α_*zz*_, while the QRCW results show a 0.04% error in α_*zz*_. In the case of RT-CC3 simulations, the LRCW results show a 0.06% error in α_*xx*_, a 0.04% error in α_*yy*_, and a 0.01% error in α_*zz*_, while the QRCW results indicate a 0.05% error in α_*yy*_ and a 0.09% error in α_*zz*_. Both of the two ramped continuous waves yield errors well below 0.1%. Importantly, QRCW requires less simulation time compared to LRCW and offers a slightly better curve fitting. As a result, the QRCW is the preferred choice, and this conclusion applies to both RT-CCSD and RT-CC3 simulations.

For the first hyperpolarizabilities, we observe larger errors in RT-CCSD results compared to LR-CCSD, as well as differences between single- and double-precision results. This outcome is reasonable, considering that we are calculating higher-order induced dipole moments. It is important to note that the *R*^2^ values in Tables 3 and 5 are identical. This is because the hyperpolarizabilities associated with second harmonic generation (SHG) and optical rectification (OR) are obtained using the same curve fitting process, with the field applied in a specific direction.

Tables 4 and 6 summarize the percentage errors of hyperpolarizability elements obtained from RT-CCSD calculations, compared to LR-CCSD. In Table 4, the largest error of 5.38% occurs in β_*zxx*_ from the RT-CCSD (dp) calculation using LRCW. By switching to the QRCW, the error in β_*zxx*_ is reduced by 82.71%, to 0.93%. The percentage errors for other elements are also substantially reduced by at least 48.81%. Moreover, the *R*^2^ values improve when using the QRCW, as seen in Table 3. Notably, for β_*zyy*_ where the applied field is perpendicular to the molecular plane, a less smooth trajectory of the second-order dipole moments leads to lower *R*^2^ values for the LRCW case. Using the QRCW recovers the quality of curve fitting, with *R*^2^ values exceeding 0.97.

**Table 4:** Percentage Errors of RT-CCSD/cc-pVDZ First Hyperpolarizabilities (au) Associated with SHG of H_2_O at 582 nm from Calculations Using LRCW and QRCW

	method	β_*zxx*_ (%)	β_*zyy*_ (%)	β_*zzz*_ (%)
LRCW	RT-CCSD (dp)	5.38	0.71	0.28
	RT-CCSD (sp)	5.06	0.75	0.26
QRCW	RT-CCSD (dp)	0.93	0.08	0.05
	RT-CCSD (sp)	2.59	0.22	0.11

Regarding precision arithmetic, the double-precision calculation with the LRCW outperforms the single-precision case for β_*zyy*_, while showing slightly worse results for β_*zxx*_ and β_*zzz*_. However, for calculations using the QRCW, the single-precision arithmetic leads to larger errors for all elements. Generally, a double-precision calculation should yield more accurate and robust results because double-precision floating-point numbers are accurate up to 15 digits, whereas single-precision numbers are accurate only up to around seven digits. In our test case, when the LRCW is used, the major error arises from the choice of ramping. This can be observed from the relatively large overall error and the poor *R*^2^ values. The difference caused by the two different precision arithmetics is not as pronounced. Neither of them produces sufficiently accurate results. However, when the QRCW is employed, the percentage error is significantly reduced due to the more gradual and smooth switch-on of the field, regardless of the chosen precision arithmetics. Consequently, the lower precision arithmetic becomes the primary factor contributing to the resulting error. This is evident in the last two rows of Table 4, where errors in single-precision calculations are more than twice those in the double-precision calculations.

A similar analysis applies to the results of hyperpolarizabilities associated with OR, as shown in Tables 5 and 6. In this case, the percentage errors originating from the LRCW are not as significant as those observed in the case of hyperpolarizabilities associated with SHG. Results from the double-precision calculations are consistently more accurate than the single-precision results. Furthermore, the QRCW continues to significantly enhance accuracy for each element, consistent with the trends observed for hyperpolarizabilities associated with SHG.

**Table 5:** RT-CCSD/cc-pVDZ and RT-CC3/cc-pVDZ First Hyperpolarizabilities (in Atomic Units) Associated with Optical Rectification (OR) of H_2_O at 582 nm Obtained from Simulations with Linear Ramped Continuous Wave (LRCW) and Quadratic Ramped Continuous Wave (QRCW) Fields^a^

	method	β_*zxx*_	β_*zyy*_	β_*zzz*_			
	LR-CCSD	–4.488	–30.485	–18.830			
LRCW	RT-CCSD (dp)	–4.532	–30.568	–18.927	0.93021	0.54362	0.89911
	RT-CCSD (sp)	–4.579	–30.624	–18.977	0.92954	0.54195	0.89921
QRCW	RT-CCSD (dp)	–4.481	–30.513	–18.848	0.99892	0.97345	0.99488
	RT-CCSD (sp)	–4.445	–30.733	–18.918	0.99169	0.97266	0.99480
LRCW	RT-CC3 (dp)	–4.292	–30.516	–18.235	0.92891	0.59673	0.89844
	RT-CC3 (sp)	–4.289	–30.529	–18.210	0.92874	0.59542	0.89839
QRCW	RT-CC3 (dp)	–4.240	–30.415	–18.146	0.99887	0.97110	0.99475
	RT-CC3 (sp)	–4.312	–30.433	–18.128	0.99273	0.97154	0.99474

a Reference values from LR-CCSD calculations using CFOUR are provided. The *R*^2^ values, indicating the quality of curve fitting, are displayed in the last three columns.

**Table 6:** Percentage Errors of RT-CCSD/cc-pVDZ First Hyperpolarizabilities (au) Associated with OR of H_2_O at 582 nm from Calculations Using LRCW and QRCW

	method	β_*zxx*_ (%)	β_*zyy*_ (%)	β_*zzz*_ (%)
LRCW	RT-CCSD (dp)	0.98	0.27	0.51
	RT-CCSD (sp)	2.03	0.46	0.78
QRCW	RT-CCSD (dp)	0.16	0.09	0.10
	RT-CCSD (sp)	0.96	0.81	0.47

In the context of RT-CC3 calculations, even though reference values are unavailable for direct comparison, the impact of replacing the LRCW with the QRCW is evident from the substantial increase in *R*^2^ values. The excellent *R*^2^ values observed in the QRCW RT-CC3 calculations further reinforce the notion that our implementation serves as a viable tool for calculating dynamic polarizability and first hyperpolarizabilities at the CC3 level, given the limitations of available alternatives.

The results presented above demonstrate the capability of the RT-CC3 method for calculating polarizabilities and first hyperpolarizabilities. Given the approximated orbital relaxation with singles in CC3, it is worthwhile to explore a comparison to orbital-optimized coupled cluster (OCC) methods where the singles cluster operators are replaced by orbital rotations. As an example, Kristiansen et al.^59^ implemented real-time (RT) time-dependent orbital-optimized second-order Møller-Plesset (TDOMP2) theory,^61^ which serves as a second-order approximation to the time-dependent orbital-optimized coupled cluster doubles (TDOCCD) method.^31,62^ TDOMP2 is further compared to RT-CC2, which is a second-order approximation to RT-CCSD. Kristiansen et al. showed that while orbital optimization does not significantly affect linear absorption spectra, it leads to a significant improvement relative to RT-CC2 theory for polarizabilities and hyperpolarizabilities. This observation also holds for complex-valued polarizabilities obtained in the presence of a static uniform magnetic field.^63^

In addition to the TDOMP2 method, Kristiansen et al. also developed the time-dependent nonorthogonal OCCD (TDNOCCD) method,^30,53^ where the orbital rotation is nonunitary, which is crucial for convergence to the FCI limit.^64,65^ To assess the performance of RT-CC3 and TDNOCCD for polarizabilities, several ten-electron systems are investigated using double-precision calculations to mitigate errors stemming from low-precision arithmetic. Table 7 presents the TDNOCCD results computed using the Hylleraas Quantum Dynamics (HyQD) software library^66^ and compares them with our RT-CC3 results. Reference values include FCI values and LR results, with RT-CCSD results included for comparison. FCI and LR-CC3 values for Ne and HF are obtained from ref (67). LR-CC3 values for other molecules are computed using CFOUR. All RT simulations employ the QRCW as the applied field with *n*_r_ = *n*_p_ = 1.

**Table 7:** Polarizabilities (in Atomic Units) of Ne, HF, H_2_O, NH_3_ and CH_4_ for Various Values of ω

Ne					
	ω = 0.1 a.u.	ω = 0.2 a.u.	ω = 0.3 a.u.	ω = 0.4 a.u.	ω = 0.5 a.u.
FCI	2.70	2.79	2.97	3.31	4.09
LR-CC3	2.71	2.80	2.98	3.32	4.10
RT-CC3	2.71	2.80	2.99	3.67	4.11
LR-CCSD	2.74	2.83	3.01	3.38	4.23
RT-CCSD	2.74	2.83	3.03	3.49	4.76
TDNOCCD	2.66	2.75	2.92	3.41	3.93

HF						
	ω = 0.1 a.u.		ω = 0.2 a.u.		ω = 0.3 a.u.	
	α_*yy*_	α_*zz*_	α_*yy*_	α_*zz*_	α_*yy*_	α_*zz*_
FCI	4.39	6.33	4.76	6.74	6.00	7.63
LR-CC3	4.39	6.34	4.77	6.76	6.03	7.64
RT-CC3	4.39	6.34	4.73	6.75	6.52	7.48
LR-CCSD	4.44	6.41	4.83	6.83	6.19	7.73
RT-CCSD	4.45	6.41	4.84	6.83	6.72	7.84
TDNOCCD	4.30	6.21	4.63	6.60	6.09	7.35

	H_2_O								
	ω = 0.0428 a.u.			ω = 0.0656 a.u.			ω = 0.1 a.u.		
	α_*xx*_	α_*yy*_	α_*zz*_	α_*xx*_	α_*yy*_	α_*zz*_	α_*xx*_	α_*yy*_	α_*zz*_
LR-CC3	8.72	9.86	9.04	8.83	9.92	9.12	9.10	10.06	9.30
RT-CC3	8.72	9.86	9.04	8.83	9.92	9.12	9.10	10.06	9.31
LR-CCSD	8.78	9.93	9.11	8.89	9.99	9.19	9.18	10.14	9.37
RT-CCSD	8.78	9.93	9.11	8.90	10.00	9.19	9.19	10.14	9.37
TDNOCCD	8.45	9.67	8.84	8.55	9.73	8.90	8.79	9.86	9.07

NH_3_						
	ω = 0.0428 a.u.		ω = 0.0656 a.u.		ω = 0.1 a.u.	
	α_*yy*_	α_*zz*_	α_*yy*_	α_*zz*_	α_*yy*_	α_*zz*_
LR-CC3	13.05	15.02	13.15	15.33	13.39	16.14
RT-CC3	13.05	15.02	13.15	15.33	13.38	15.99
LR-CCSD	13.10	15.04	13.20	15.35	13.44	16.15
RT-CCSD	13.10	15.05	13.20	15.36	13.45	16.15
TDNOCCD	12.85	14.57	12.94	14.84	13.17	15.51

CH_4_			
	ω = 0.0656 a.u.	ω = 0.1 a.u.	ω = 0.2 a.u.
LR-CC3	17.04	17.38	19.55
RT-CC3	17.04	17.37	19.34
LR-CCSD	17.05	17.39	19.55
RT-CCSD	17.05	17.39	19.58
TDNOCCD	16.86	17.19	19.22

For Ne, RT-CC3 exhibits good agreement with LR-CC3 and FCI, with errors of at most 0.67% for frequencies ranging from 0.1 to 0.3 au. However, a notable deviation from LR-CC3 and FCI results becomes apparent at a frequency of 0.4 au, which is closer to the resonance at 0.613 au. The accuracy of the result at ω = 0.5 au is expected to be even lower, as indicated by the comparison between LR-CCSD and RT-CCSD. In fact, the RT-CC3 result at ω = 0.5 au is closer to the reference value, which is likely coincidental. To assess the quality of curve fitting, *R*^2^ values are compared for different frequencies. The *R*^2^ values for ω = 0.3 au, ω = 0.4 au, and ω = 0.5 au are 0.99651, 0.99318, and 0.98151, respectively. These values decrease with higher frequencies. While the result at ω = 0.5 au is “accurate,” it is somewhat less reliable than the results for lower frequencies.

A similar trend is observed for HF. RT-CC3 regains accuracy compared to LR-CC3 and FCI at frequencies of 0.1 and 0.2 au. At a frequency of 0.3 au, which is quite close to the resonance at 0.383 au, the accuracy decreases, resulting in percentage errors of 8.13 and 2.09% for α_*yy*_ and α_*zz*_, respectively. Corresponding *R*^2^ values are 0.97622 and 0.97835, respectively. The slightly larger error of α_*yy*_ is related to the symmetry of HF. The first excitation involves one of the lone pair electrons of Fluoride and the σ* orbital. Since the lone pair electrons align with the *y*-axis, the α_*yy*_ component is more relevant to the transient and therefore exhibits a larger percentage error. The observed pattern in CC3 results aligns with that in the CCSD results.

For H_2_O, selected frequencies are all well below the resonance at 0.277 au. RT-CC3 values consistently align with LR-CC3, with only a 0.11% error observed in α_*zz*_ at ω = 0.1 *au*. In the case of NH_3_, RT-CC3 results match LR-CC3 values for all frequencies chosen, which are all below the resonance at 0.236 au. The exception is α_*zz*_ at ω = 0.1 au where a 0.93% error occurs. This small discrepancy contrasts with the agreement between LR-CCSD and RT-CCSD at the same frequency. Notably, the lone pair electrons of nitrogen that are significant to the lowest excitation level are aligned with the *z*-axis.

A discrepancy is also seen in the results for CH_4_ at the frequency of 0.2 au, whereas the resonance occurs at 0.38 au. RT-CC3 results show a 1.07% deviation from LR-CC3, compared to a mere 0.15% discrepancy in the CCSD case. The corresponding *R*^2^ values for these two less accurate results, α_*zz*_ of NH_3_ and the polarizability of CH_4_, are 0.99823 and 0.99574, respectively. These values are smaller than those of the other polarizability values, which are all above 0.9999.

To further explore the differences between RT-CCSD and RT-CC3 in these cases, we calculated two additional LR-CCSD/LR-CC3 polarizabilities at different frequencies and performed polynomial regression with five data points. This analysis reveals the relationship between increasing polarizabilities and frequency. In addition to the values listed in Table 7, α_*zz*_ of NH_3_ is calculated at a frequency of 0.025 au, yielding LR-CC3 and LR-CCSD results of 14.88 and 14.90 au, respectively. At a frequency of 0.085 au, α_*zz*_ values are 15.72 and 15.74 au for LR-CC3 and LR-CCSD, respectively. Two more frequencies, 0.0428 and 0.15 au, are selected for CH_4_. RT-CC3 and RT-CCSD results at ω = 0.0428 *au* are 16.89 and 16.91 au, respectively. At ω = 0.15 au, the corresponding results are 18.20 and 18.21 au, respectively.

The polarizability can be written as a Taylor expansion containing only even orders of the frequency ω,^68^
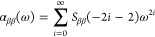
46which converges for frequencies below the first excitation energy. The coefficients *S*_ββ_(*i*) are oscillator-strength sum rules, also known as Cauchy moments,^69^ and contain a wealth of information about molecular properties.^68^ Hättig et al. have studied the Cauchy moments using LR-CCS, LR-CC2, and LR-CCSD theory.^69^ Here, we were able to obtain the Cauchy moments using theories at the level of CCSD and CC3, both LR theory and RT methods for comparison. The Taylor expansion was truncated after the third term (ω^4^) for both LR and RT results.

**Figure 4:**
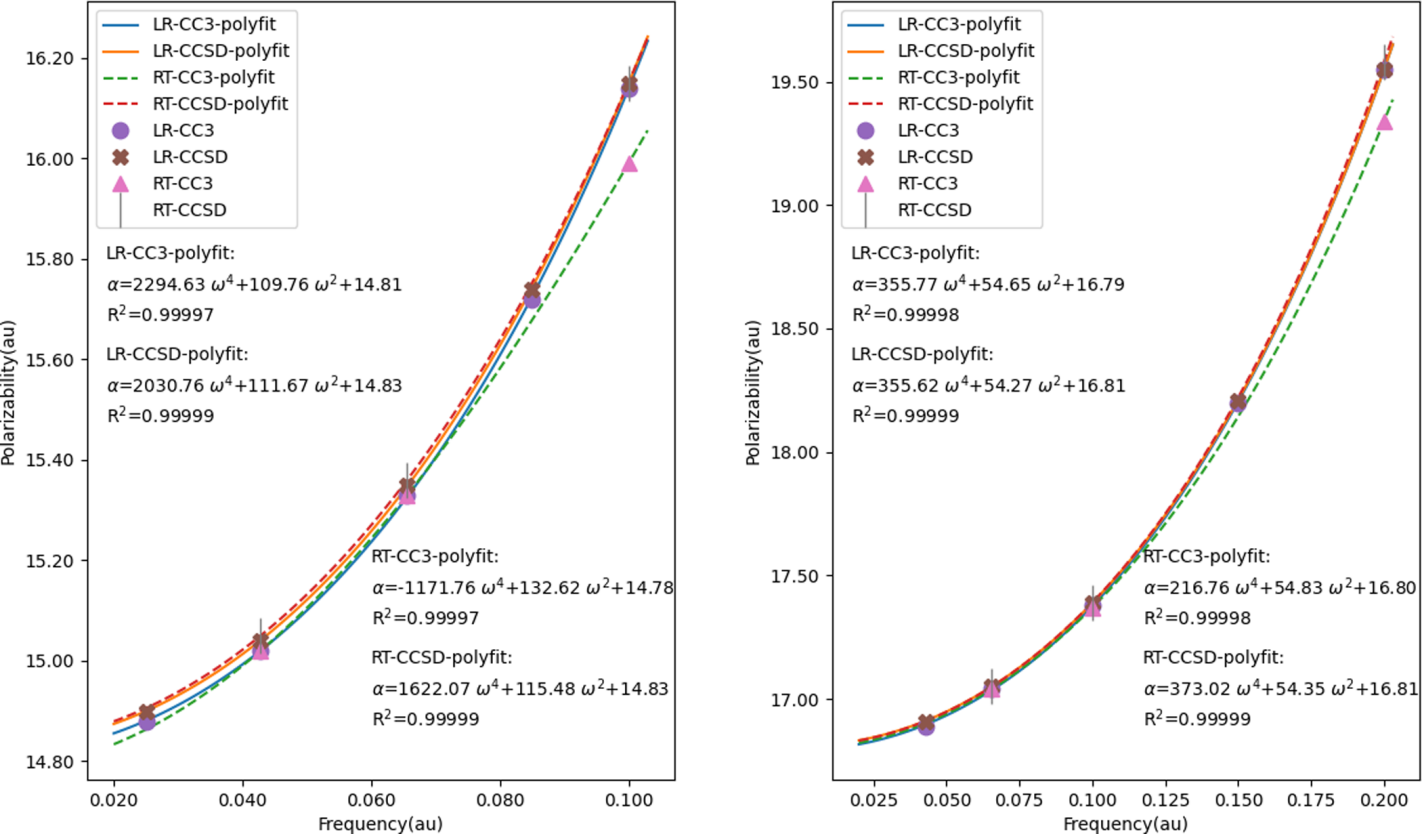
Dispersion of α_*zz*_ for NH_3_ (left) and polarizabilities for CH_4_ (right), calculated using CC3 and CCSD methods. Polynomial regression curves are depicted, along with the resulting functions and *R*^2^ values as annotations on the figures.

As shown in Figure 4, polynomial regression for LR-CCSD and LR-CC3 results closely aligns with data points for all four data sets, with *R*^2^ values exceeding 0.9999. Moreover, the coefficients of ω^4^ from LR-CC3 data are larger than those from LR-CCSD data for both NH_3_ and CH_4_. The polynomial regression results suggest that CC3 polarizabilities increase slightly faster with frequency compared to CCSD polarizabilities. The impact of frequency moving toward the resonance on polarizability results becomes evident earlier in the frequency range for CC3 compared to CCSD in these test cases, potentially explaining the disagreement observed for polarizability values of NH_3_ and CH_4_. For the RT-CCSD and RT-CC3 results, the coefficients of ω^4^ from RT-CC3 data are smaller than those from RT-CCSD data. The *R*^2^ values are still close to 1.0, however, the regression is largely affected by the polarizabilities near resonance, especially involving only three data points. The coefficients of ω^4^ deviates from LR results, while the ones of ω^2^ and the constant terms do not deviate as much. More data points in the frequency range that are away from the resonance should resolve the deviation as the polarizabilites align well with the ones from LR calculations. In the meantime, *S*_ββ_(−2*i* – 2) are obtained from the regression according to eq 46. To confirm the accuracy at low frequencies, the *S*_ββ_(−2) coefficient is compared to the static polarizability. For NH_3_, the static polarizabilities obtained from LR-CCSD and LR-CC3 are 14.83 and 14.81 au respectively. The RT-CC3 result has a 0.2% relative difference compared to its reference value, while the RT-CCSD error is effectively zero. For CH_4_, the static polarizabilities obtained from LR-CCSD and LR-CC3 are 16.80 and 16.79 respectively. All the *S*_ββ_(−2) results align well with the reference value with the relative difference smaller than 0.1%. We have shown that the method is capable of calculating Cauchy moments conveniently using theories at the level of CCSD and CC3, although more data points will be needed to obtain accurate values of Cauchy moments with larger *i*.

The impact of orbital optimization is explored by comparing TDNOCCD with RT-CCSD and higher levels of theory. As previously mentioned, TDNOCCD differs from RT-CCSD by substituting singles with a nonunitary orbital rotation, where the rotation parameters are time-dependent. Orbital-optimized CC methods have advantages in multielectron ionization dynamics, chemical bond breaking, response theory, and more.^30,31,41,44,53,61,64,65^ Explicit orbital optimization also enhances the stability of real-time simulations when systems are subjected to strong external fields, and the ground state no longer dominates because the time-dependent reference determinant tends to be close or identical to the Brueckner determinant.^70^

In our test cases, TDNOCCD is initially compared to RT-CCSD by assessing its differences from LR-CCSD. The data presented in Table 7 indicate that TDNOCCD results exhibit relative differences ranging from 0.89 to 7.09%, with an average difference of 2.98% across all frequencies and molecules, compared to LR-CCSD. The most significant difference arises from the polarizability of Ne at ω = 0.5 *au*. However, this TDNOCCD result is actually closer to LR-CCSD than RT-CCSD for this specific value. Unlike the RT methods, the frequency dependence of relative differences in TDNOCCD is not as pronounced. It is evident that substantial differences are present not only in high-frequency results but also in low-frequency outcomes, which are distant from resonances. The primary factor contributing to this divergence between TDNOCCD and RT-CCSD, in comparison with LR-CCSD, is the orbital optimization.

Next, TDNOCCD results and RT-CC3 are compared to LR-CC3. As documented in ref (67), LR-CC3 can be taken as a reference value considering its high accuracy compared to FCI, although this choice gives a slight bias toward methods without orbital optimization. Theoretically, RT-CC3 should exactly reproduce LR-CC3 (up to numerical differentiation, accuracy of the integrator, etc.) by adding more ramping cycles. Except for a few cases (Ne at ω = 0.4 and 0.5 au, HF at ω = 0.3 au, NH_3_ at ω = 0.1 au, and CH_4_ at ω = 0.2 au), RT-CC3 results match LR-CC3. TDNOCCD, however, deviates from LR-CC3/RT-CC3 by at least 1.03% and up to 3.41%, with an average deviation of 2.14%. As these polarizability results are unaffected by proximity to resonances, the deviation stems from the distinct treatments of orbital optimization and the inclusion or exclusion of triples.

In cases where RT-CC3 exhibits significant percentage errors compared to LR-CC3, TDNOCCD may be closer or farther from LR-CC3. When RT-CC3 overestimates polarizability of Ne (ω = 0.4 au) and α_*yy*_ of HF (ω = 0.3 au) by 10.54 and 8.13%, respectively, TDNOCCD yields smaller values closer to LR-CC3 due to orbital optimization, underestimating these polarizability values. When RT-CC3 underestimates polarizabilities, the even smaller values from TDNOCCD result in a larger difference from LR-CC3.

#### 4.2.3 *G′* Tensor and Quadratic Response Function

**Figure 5:**
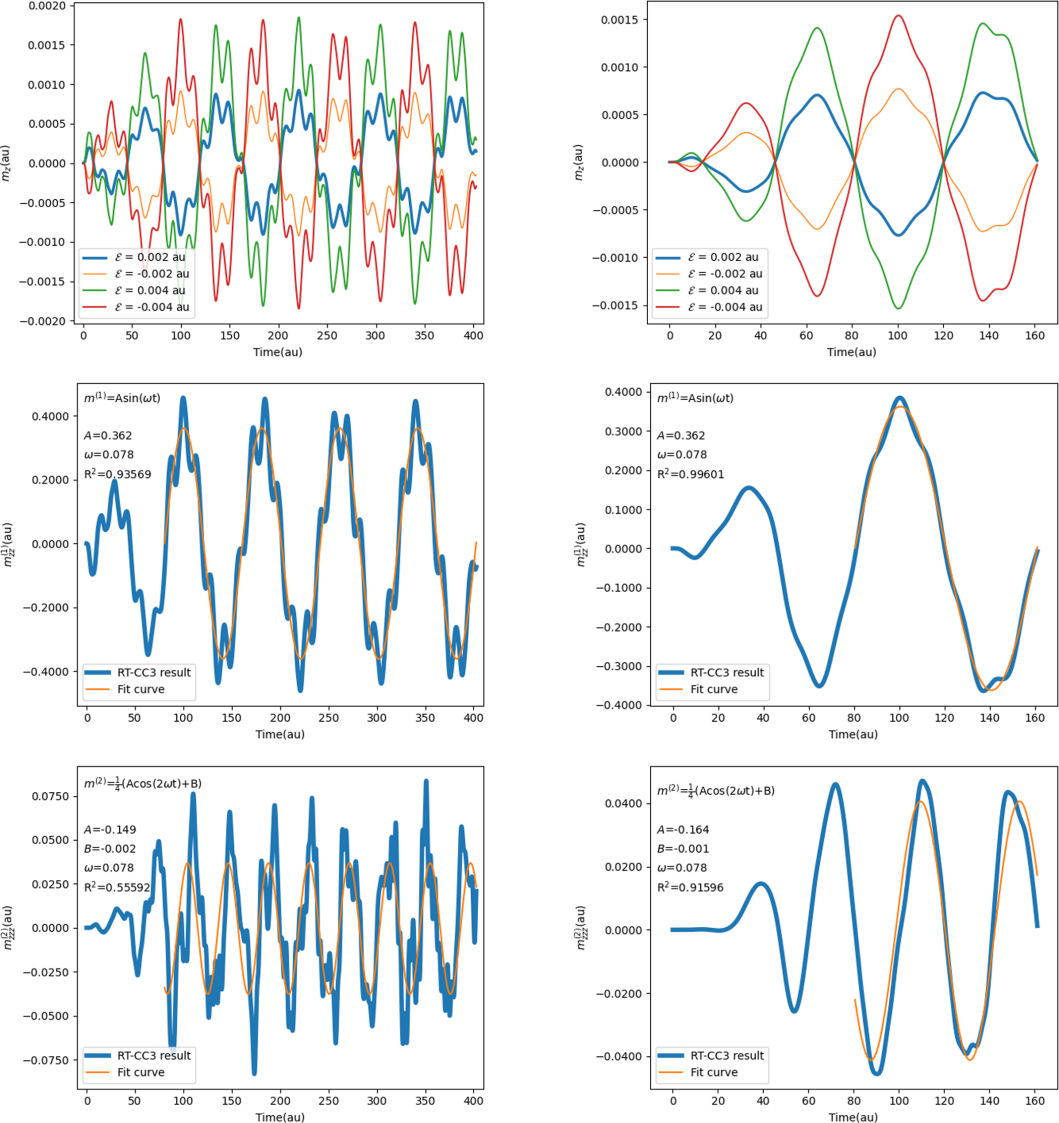
RT-CC3/cc-pVDZ (dp) results of H_2_ dimer obtained from four simulations with field strengths of 0.002, −0.002, 0.004, and −0.004 au. The left column displays the LRCW results, including the *z* component of the induced magnetic dipole moment and the corresponding first-order and second-order dipole moments with their fitted curve. The right column presents the QRCW results.

With our RT-CC implementation, we calculate the *G′* tensor of the H_2_ dimer using RT-CCSD and RT-CC3, both in single- and double-precision arithmetics. We employ both the LRCW and the QRCW for comparison purposes. As illustrated in Figure 5, we utilize the induced magnetic dipole moments from applied fields of varying strengths to compute the first-order magnetic dipole moments through the finite difference method, similar to the procedure for calculating polarizabilities. In this case, *G*_*zz*_*′* can be obtained from the magnetic dipole moment induced by the field applied in the *z* direction, with its value represented by the amplitude of the fitted curve.

As per Table 8, no distinction is observed between single- and double-precision results in the RT-CCSD calculations. The *G′* tensor elements exhibit identical values for both the LRCW and the QRCW cases, with the distinction lying solely in the *R*^2^ values. Notably, the QRCW significantly enhances curve fitting quality, aligning with the conclusion drawn in Section 4.2.2. Upon examining the example results in Figure 5, it is evident that the induced magnetic dipole moment curves are less smooth compared to the induced electric dipole moment curves discussed in the previous section. Particularly in the LRCW instance, a curvilinear trajectory of the dipole is observed. Although this irregular shape remains approximately periodic, it adversely affects curve fitting. A minor distortion is observed in the QRCW example, which has a lesser impact on curve fitting. Table 9 presents the RT-CC3 results. Analogous to RT-CCSD, the disparities between single- and double-precision results are inconsequential. The QRCW enhances overall *R*^2^ values and provides more reliable outcomes. Hence, the RT-CC3 method combined with QRCW is a viable approach for computing the *G′* tensor and subsequently optical rotation.

**Table 8:** RT-CCSD/cc-pVDZ *G′* Tensor Elements (in Atomic Units) of H_2_ Dimer at 582 nm Obtained Using Single- and Double-Precision Computations, with Linear Ramped Continuous Wave (LRCW) and Quadratic Ramped Continuous Wave (QRCW) Fields^a^

	*G′*	RT-CCSD (dp)	*R*_*x*_^2^(dp)	*R*_*y*_^2^(dp)	*R*_*z*_^2^(dp)
LRCW	(*G*_*xx*_*′*, *G*_*yx*_*′*, *G*_*zx*_*′*)	(−0.387, −0.097, 0.000)	0.93726	0.93911	0
	(*G*_*xy*_*′*, *G*_*yy*_*′*, *G*_*zy*_*′*)	(0.058, 0.013, 0.000)	0.88177	0.86203	0
	(*G*_*xz*_*′*, *G*_*yz*_*′*, *G*_*zz*_*′*)	(0.000, 0.000, 0.362)	0	0	0.93669

	*G′*	RT-CCSD (sp)	*R*_*x*_^2^(sp)	*R*_*y*_^2^(sp)	*R*_*z*_^2^(sp)
LRCW	(*G*_*xx*_*′*, *G*_*yx*_*′*, *G*_*zx*_*′*)	(−0.387, −0.097, 0.000)	0.93727	0.93911	0
	(*G*_*xy*_*′*, *G*_*yy*_*′*, *G*_*zy*_*′*)	(0.058, 0.013, 0.000)	0.88179	0.86205	0
	(*G*_*xz*_*′*, *G*_*yz*_*′*, *G*_*zz*_*′*)	(0.000, 0.000, 0.362)	0	0	0.93669

	*G′*	RT-CCSD (dp)	*R*_*x*_^2^(dp)	*R*_*y*_^2^(dp)	*R*_*z*_^2^(dp)
QRCW	(*G*_*xx*_*′*, *G*_*yx*_*′*, *G*_*zx*_*′*)	(−0.387, −0.097, 0.000)	0.99956	0.99956	0
	(*G*_*xy*_*′*, *G*_*yy*_*′*, *G*_*zy*_*′*)	(0.058, 0.013, 0.000)	0.99915	0.99896	0
	(*G*_*xz*_*′*, *G*_*yz*_*′*, *G*_*zz*_*′*)	(0.000, 0.000, 0.362)	0	0	0.99597

	*G′*	RT-CCSD (sp)	*R*_*x*_^2^(sp)	*R*_*y*_^2^(sp)	*R*_*z*_^2^(sp)
QRCW	(*G*_*xx*_*′*, *G*_*yx*_*′*, *G*_*zx*_*′*)	(−0.387, −0.097, 0.000)	0.99956	0.99956	0
	(*G*_*xy*_*′*, *G*_*yy*_*′*, *G*_*zy*_*′*)	(0.058, 0.013, 0.000)	0.99915	0.99896	0
	(*G*_*xz*_*′*, *G*_*yz*_*′*, *G*_*zz*_*′*)	(0.000, 0.000, 0.362)	0	0	0 0.99597

a The *R*^2^ values, indicating the quality of curve fitting, are displayed in the last three columns.

**Table 9:** RT-CC3/cc-pVDZ *G′* Tensor Elements (in Atomic Units) of H_2_ Dimer at 582 nm Obtained Using Single- and Double-Precision Computations, with Linear Ramped Continuous Wave (LRCW) and Quadratic Ramped Continuous Wave (QRCW) Fields^a^

	*G′*	RT-CC3 (dp)	*R*_*x*_^2^(dp)	*R*_*y*_^2^(dp)	*R*_*z*_^2^(dp)
LRCW	(*G*_*xx*_*′*, *G*_*yx*_*′*, *G*_*zx*_*′*)	(−0.387, −0.097, 0.000)	0.93654	0.93842	0
	(*G*_*xy*_*′*, *G*_*yy*_*′*, *G*_*zy*_*′*)	(0.058, 0.013, 0.000)	0.87831	0.85893	0
	(*G*_*xz*_*′*, *G*_*yz*_*′*, *G*_*zz*_*′*)	(0.000, 0.000, 0.362)	0	0	0.93569

	*G′*	RT-CC3 (sp)	*R*_*x*_^2^(sp)	*R*_*y*_^2^(sp)	*R*_*z*_^2^(sp)
LRCW	(*G*_*xx*_*′*, *G*_*yx*_*′*, *G*_*zx*_*′*)	(−0.387, −0.097, 0.000)	0.93654	0.93842	0
	(*G*_*xy*_*′*, *G*_*yy*_*′*, *G*_*zy*_*′*)	(0.058, 0.013, 0.000)	0.87831	0.85893	0
	(*G*_*xz*_*′*, *G*_*yz*_*′*, *G*_*zz*_*′*)	(0.000, 0.000, 0.362)	0	0	0.93569

	*G′*	RT-CC3 (dp)	*R*_*x*_^2^(dp)	*R*_*y*_^2^(dp)	*R*_*z*_^2^(dp)
QRCW	(*G*_*xx*_*′*, *G*_*yx*_*′*, *G*_*zx*_*′*)	(−0.387, −0.097, 0.000)	0.99955	0.99955	0
	(*G*_*xy*_*′*, *G*_*yy*_*′*, *G*_*zy*_*′*)	(0.058, 0.013, 0.000)	0.99911	0.99891	0
	(*G*_*xz*_*′*, *G*_*yz*_*′*, *G*_*zz*_*′*)	(0.000, 0.000, 0.362)	0	0	0.99602

	*G′*	RT-CC3 (sp)	*R*_*x*_^2^(sp)	*R*_*y*_^2^(sp)	*R*_*z*_^2^(sp)
QRCW	(*G*_*xx*_*′*, *G*_*yx*_*′*, *G*_*zx*_*′*)	(−0.387, −0.097, 0.000)	0.99955	0.99955	0
	(*G*_*xy*_*′*, *G*_*yy*_*′*, *G*_*zy*_*′*)	(0.058, 0.013, 0.000)	0.99911	0.99891	0
	(*G*_*xz*_*′*, *G*_*yz*_*′*, *G*_*zz*_*′*)	(0.000, 0.000, 0.362)	0	0	0.99601

a The *R*^2^ values, indicating the quality of curve fitting, are displayed in the last three columns.

With the same data set, it is also possible to extract quadratic response functions of the form ⟨⟨; , ⟩⟩_ω,ω*′*_ using the second-order induced magnetic dipole moments. Such response functions describe magnetic-dipole second harmonic generation for ω*′* = ω, while for ω*′* = −ω they are related to Verdet’s constant and magnetic optical rotation.^71,72^ The latter can, of course, also be obtained directly from the polarizability in a finite magnetic field.^63^ As shown in Figure 5, ⟨⟨; ,  and ⟨⟨; , ⟩⟩_ω,−ω_ can be obtained as the amplitude and phase of the fitted curve, respectively, and RT-CCSD and RT-CC3 results are listed in Table 10. No significant difference is observed between single- and double-precision results for both of the response functions, although the *R*^2^ value of the single-precision calculation is slightly lower. It is obvious that the quality of the curve fitting is not sufficient to provide robust results, especially for the LRCW cases. Compared to the hyperpolarizability results obtained from  in Tables 3 and 5, the second-order response of the magnetic dipole moments to the external electric field is much weaker. The results are more sensitive to the choice of the external field, the length of the propagation, the numerical differentiation, etc. For example, the relative difference between the LRCW and the QRCW results of RT-CC3 (dp) ⟨⟨; , ⟩⟩_ω,ω_ (9.15%) is larger than the relative difference of β_*zzz*_ (SHG) (0.49%). QRCW with extra ramped cycles is assumed to be necessary and helpful to improve the quality of the curve fitting and provide reliable results. In that case, the RT-CC framework would be practical for calculating ⟨⟨; , ⟩⟩ straightforwardly.

**Table 10:** RT-CCSD/cc-pVDZ and RT-CC3/cc-pVDZ ⟨⟨*m̂*_*z*_; μ̂_*z*_, μ̂_*z*_⟩⟩ (in Atomic Units) of H_2_ Dimer at 582 nm Obtained from Simulations with Linear Ramped Continuous Wave (LRCW) and Quadratic Ramped Continuous Wave (QRCW) Fields^a^

	method			*R*^2^
LRCW	RT-CCSD (dp)	–0.147	–0.001	0.51509
	RT-CCSD (sp)	–0.147	–0.001	0.47038
QRCW	RT-CCSD (dp)	–0.161	–0.001	0.91640
	RT-CCSD (sp)	–0.161	–0.001	0.89186
LRCW	RT-CC3 (dp)	–0.149	–0.002	0.55592
	RT-CC3 (sp)	–0.149	–0.002	0.54161
QRCW	RT-CC3 (dp)	–0.164	–0.001	0.91596
	RT-CC3 (sp)	–0.163	–0.002	0.87705

a The *R*^2^ values, reflecting the quality of curve fitting, are displayed in the last column.

## 5 Conclusions

The RT-CC3 method has been implemented with additional single-precision and GPU options. The working equations of RT-CC3 in the closed-shell and spin-adapted formalism are provided, with considerations of optimizing the performance in terms of reducing the number of higher-order tensor contractions. The implementation has been validated through the calculation of the absorption spectrum of H_2_O in both single- and double-precision. Numerical experiments have also been conducted with water clusters to test the computational cost of RT-CC3 simulations. It has been found that the use of GPUs can significantly speed up calculations by up to a factor of 17 due to the computational power they provide for tensor contractions. The acceleration gained from utilizing either GPUs or single-precision arithmetic needs to be observed significantly within a relatively large system (e.g., 72 molecular orbitals). To achieve the theoretical speedup, a much larger system is needed, however, optimization of memory allocation will also need to be taken into account because of the limited memory available on GPUs and the overhead of data migration. With the promising results of our Python implementation, exploring a productive-level code is worthwhile, especially for making the RT-CC3 method, which scales as (*N*^7^), feasible for large system/basis set and/or long RT propagations.

For the calculation of optical properties, we have demonstrated that RT-CC3 is a feasible tool to obtain dynamic polariazabilities, first hyperpolarizabilities, and the *G′* tensor with good agreement to LR-CC3 and a reasonable computational cost. The type of applied field, the precision arithmetic, and the level of theory were tested with H_2_O and H_2_ dimer. It has been demonstrated through all test cases, including our new RT-CC3 method, that the QRCW can substantially improve curve fitting and requires only two optical cycles for propagation. Especially for first hyperpolarizabilites, the curve of the second-order dipole moments from LRCW calculation has “discontinuities” in some places, leading to a large error and a low *R*^2^ value for curve fitting. The QRCW is required here to obtain reliable results. The same is found in *G′* tensor results, where some shifts appear in the curve of the induced magnetic dipole moments from LRCW calculations but not the QRCW ones. Regarding the single-precision calculations, no discrepancy is found in the polarizabilities and *G′* tensor elements that are associated with the first derivative of electric and magnetic dipole moments, respectively. A significant difference, however, is found in the first hyperpolarizabilities. Although the QRCW can still reduce the error compared to the LRCW, single-precision results remain less accurate compared to double-precision results. With the same set of induced magnetic dipole moments for obtaining the *G′* tensor, we can also extract the quadratic response function ⟨⟨; , ⟩⟩_ω,ω′_, although QRCW with extra ramped cycles is assumed to be needed for more accurate results. These conclusions hold for both RT-CCSD and RT-CC3.

Additionally, ten-electron systems including Ne, HF, H_2_O, NH_3_ and CH_4_ are used to test the calculation of polarizabilities with RT-CCSD, RT-CC3, and particularly TDNOCCD. It has been observed that the accuracy drops significantly when the frequency is closer to the resonance, while for the small frequencies, RT-CC3 matches LR-CC3 and FCI with errors less than 0.1%. The trend of the error of RT-CC3 is consistent with RT-CCSD for most cases, except for the two values with the highest chosen frequencies of NH_3_ and CH_4_. We have shown that the CC3 polarizabilities increase slightly faster when the frequency moves toward resonance, which may lead to a larger error. The TDNOCCD results show that the explicit orbital optimization lowers the polarizability values compared to RT-CCSD, where the only difference in these two methods is the orbital optimization. Compared to RT-CC3, TDNOCCD results are closer to LR-CC3/FCI results when RT-CC3 largely overestimates the results, otherwise, RT-CC3 yields more accurate results.

## Appendix: Derivation of the RT-CC3 Equations

Assuming a closed-shell spin-restricted reference determinant, the Hamiltonian can be written in the canonical basis as

A1

A2

A3

The *T*_1_-transformed Hamiltonian is (dropping the reference to time dependence for convenience):

A4

where *F* = exp(−*T̂*_1_) *F̂* exp(*T̂*_1_) and analogously for *U* and *V*. We assume that *V̂* is a one-electron operator. β is the field strength.

The time-dependent CCSDT Lagrangian can be written as

A5

To obtain the CC3 Lagrangian we now apply the following rules regarding order in the fluctuation potential *Û*,

and *neglect all terms above fourth order in the CCSDT Lagrangian*:

A6

The EOMs can now be obtained from the Euler-Lagrange equations

A7

where *z* denotes the cluster amplitudes *t* and λ.

Setting *z* = λ_0_, we find

A8

Setting *z* = *t*_0_, we find

A9

which means that we may choose λ_0_ = 1.

The remaining *T̂* equations read (obtained by setting *z* = λ_μ_*i*__, *i* = 1, 2, 3):

A10

A11

A12

and the Λ̂ equations read (obtained by setting *z* = *t*_μ_*i*__, *i* = 1, 2, 3):

A13

A14

A15

Note that

A16

The right-hand sides of the singles and doubles equations can be separated into CCSD components and triples specific terms. To derive the spin-adapted expression of the amplitude equations, we write the singles, doubles, and triples cluster operators as

A17

A18

and

A19

respectively, where *i*, *j*, *k*,... are occupied orbitals, *a*, *b*, *c*,... are virtual orbitals, and the unitary group generators are defined as

A20

with *p*, *q* being molecular orbitals. By inserting this form of *T̂* amplitudes and the similar form for the Λ̂ amplitudes into the triples specific terms, the spin-adapted expression can be written as

A21

in the *T̂*_1_ equation,

A22

in the *T̂*_2_ equation,

A23

in the Λ̂_1_ equation, and

A24

in the Λ̂_2_ equation. In the time-independent case, the triples can be calculated as

A25

and

A26

where *v*_*pq*_ is the matrix element of the perturbation operator *V̂*. We emphasize that, in the perturbed case (i.e., non-zero β*V*), the triples cannot be computed via a closed expression in terms of the *T̂*_1_ and *T̂*_2_ amplitudes, but must be computed iteratively.

The one- and two-electron integrals, *f*_*pq*_ and ⟨*pq*|*rs*⟩, are extracted from the one- and two-electron component of the Hamiltonian, respectively, as

A27

where {*E*_*pq*_} denotes the normal ordering unitary-group generator. *L*_*pqrs*_ is defined as

A28

*f̃*_*pq*_, ⟨*pq*|~*rs*⟩ and *L̃*_*pqrs*_ are components of the *T*_1_-transformed Hamiltonian. The permutation operators are defined as

A29

and

A30

The explicit formula of the additional terms involving triples in the one-electron density can be written as

A31

A32

and

A33

## Supporting Information Available

Atomic coordinates of all molecular test cases (PDF)
