# Supplementary material for: Real-Time Coupled Cluster Theory with Approximate Triples
Source: J Phys Chem A. 2025 Feb 8;129(7):1908–27. doi: 10.1021/acs.jpca.4c08499 (PMC11848932; doi:10.1021/acs.jpca.4c08499)
Supplement: Supplementary file 1 — jp4c08499_si_001.pdf [file jp4c08499_si_001.pdf]

Real-Time Coupled Cluster Theory with Approximate Triples

Zhe Wang,† Håkon Emil Kristiansen,‡ Thomas Bondo Pedersen,‡ and T. Daniel Crawford*,†

†*Department of Chemistry, Virginia Tech, Blacksburg, VA 24061, USA*

‡ *Hylleraas Centre for Quantum Molecular Sciences, Department of Chemistry, University of Oslo, P.O. Box 1033 Blindern, N-0315 Oslo, Norway*

E-mail: crawdad@vt.edu

Molecular geometries

H_2O monomer (Bohr)

```
O 0.0000000000  0.0000000000 -0.1432258275
H 0.0000000000 -1.6380369656  1.1365489094
H 0.0000000000  1.6380369656  1.1365489094
```

H_2O dimer (Bohr)

```
O -2.8661644063 -0.1653553494  0.1406596673
H -1.0748851518  0.1278214558 -0.1769942495
H -3.7141722361  1.0872074732 -0.8867321672
O  2.6264709777  0.1816018234 -0.1439004684
H  3.0097525830 -1.5752527068 -0.5064248249
H  2.8656934862  0.3299412931  1.6689714146
```

H_2O trimer (Bohr)

```
O -2.7901834256 -1.1967418445  0.1698537525
H -3.7489330735 -1.4117507704 -1.3759977570
H -2.1682945792  0.5551889874  0.0944128906
O  2.4583562203 -1.8378202873  0.2122730854
H  3.0673681577 -2.6954017948 -1.2873471197
H  0.6394366331 -2.2195689067  0.2759689288
O  0.3904216732  2.9850604593 -0.2256467031
H  1.6073104931  1.5786082232 -0.1726868647
H  0.7085663149  3.9236448086  1.3156354427
```

H_2O tetramer (Bohr)

```
O -3.6457942233 -0.1219948910  0.2112107604
H -4.7080861934  0.0139560240 -1.2743432426
H -2.5095181169  1.3606879643  0.1081588210
O  0.1052152855 -3.7480907392  0.2365851514
H -1.4084001979 -2.6502316722  0.2899060536
H -0.1092364578 -4.7741128769 -1.2649877359
O -0.0537678204  3.6180254701 -0.2399721153
H  0.1561587878  4.6568968597  1.2534025868
H  4.7182051722 -0.1528122610  1.3660080628
O  3.7022589144 -0.0050452889 -0.1504798111
H  1.4602282658  2.5202081604 -0.2784037147
```

```
H  2.5592136957 -1.4846634281 -0.0873860598
```

HF (Bohr)

```
H 0.0000000 0.0000000 0.0000000
F 0.0000000 0.0000000 1.7328795
```

H_2O (Bohr)

```
O 0.0000000000  0.0000000000 -0.1239093563
H 0.0000000000  1.4299372840  0.9832657567
H 0.0000000000 -1.4299372840  0.9832657567
```

NH_3 (Bohr)

```
N  0.0000  0.0000  0.2010
H  0.0000  1.7641 -0.4690
H  1.5277 -0.8820 -0.4690
H -1.5277 -0.8820 -0.4690
```

CH_4 (Bohr)

```
C  0.0000  0.0000  0.0000
H  1.2005  1.2005  1.2005
H -1.2005 -1.2005  1.2005
H -1.2005  1.2005 -1.2005
H  1.2005 -1.2005 -1.2005
```

H_2 dimer (Bohr)

```
H -0.7086472998  1.4172945997 -0.6137065639
H  0.0000000000  1.4172945997  0.6137065639
H  0.0000000000 -1.4172945997  0.6137065639
H  0.7086472998 -1.4172945997 -0.6137065639
```
